# Cathepsin D expression level affects alpha-synuclein processing, aggregation, and toxicity *in vivo*

**DOI:** 10.1186/1756-6606-2-5

**Published:** 2009-02-09

**Authors:** Valerie Cullen, Maria Lindfors, Juliana Ng, Anders Paetau, Erika Swinton, Piotr Kolodziej, Heather Boston, Paul Saftig, John Woulfe, Mel B Feany, Liisa Myllykangas, Michael G Schlossmacher, Jaana Tyynelä

**Affiliations:** 1 Center for Neurologic Diseases, Department of Neurology, Brigham & Women's Hospital, Harvard Medical School, Boston, Massachusetts, USA; 2 Institute of Biomedicine/Biochemistry, Helsinki University, Helsinki, Finland; 3 Folkhälsan Institute of Genetics, Biomedicum Helsinki, Helsinki, Finland; 4 Department of Pathology, Haartman Institute, Helsinki University, Helsinki, Finland; 5 Department of Pathology, Brigham & Women's Hospital, Harvard Medical School, Boston, Massachusetts, USA; 6 Biochemical Institute, University of Kiel, Kiel, Germany; 7 Division of Neuroscience, Ottawa Health Research Institute, Ottawa, Canada; 8 Department of Pathology, University of Ottawa, Ottawa, Canada; 9 Current affiliation : Link Medicine Corp., Cambridge, Massachusetts, USA

## Abstract

**Background:**

Elevated *SNCA *gene expression and intracellular accumulation of the encoded α-synuclein (aSyn) protein are associated with the development of Parkinson disease (PD). To date, few enzymes have been examined for their ability to degrade aSyn. Here, we explore the effects of *CTSD *gene expression, which encodes the lysosomal protease cathepsin D (CathD), on aSyn processing.

**Results:**

Over-expression of human *CTSD *cDNA in dopaminergic MES23.5 cell cultures induced the marked proteolysis of exogenously expressed aSyn proteins in a dose-dependent manner. Unexpectedly, brain extractions, Western blotting and ELISA quantification revealed evidence for reduced levels of soluble endogenous aSyn in *ctsd *knock-out mice. However, these CathD-deficient mice also contained elevated levels of insoluble, oligomeric aSyn species, as detected by formic acid extraction. In accordance, immunohistochemical studies of *ctsd*-mutant brain from mice, sheep and humans revealed selective synucleinopathy-like changes that varied slightly among the three species. These changes included intracellular aSyn accumulation and formation of ubiquitin-positive inclusions. Furthermore, using an established *Drosophila *model of human synucleinopathy, we observed markedly enhanced retinal toxicity in *ctsd*-null flies.

**Conclusion:**

We conclude from these complementary investigations that: one, CathD can effectively degrade excess aSyn in dopaminergic cells; two, *ctsd *gene mutations result in a lysosomal storage disorder that includes microscopic and biochemical evidence of aSyn misprocessing; and three, CathD deficiency facilitates aSyn toxicity. We therefore postulate that CathD promotes 'synucleinase' activity, and that enhancing its function may lower aSyn concentrations *in vivo*.

## Background

α-Synuclein (aSyn) is a cytosolic and presynaptic protein strongly implicated in the pathogenesis of neurodegenerative disorders. Point mutations in the corresponding gene, *SNCA*, as well as over-expression of the wild-type variant due to locus multiplication, cause autosomal-dominant forms of Parkinson disease (PD) [1-3]. Furthermore, accumulation of aggregated, insoluble aSyn is a hallmark of many other neurodegenerative diseases, including sporadic PD, dementia with Lewy bodies (DLB), multiple system atrophy (MSA), the Lewy body variant of Alzheimer's disease, and *PANK2*-linked neurodegeneration. Collectively, these disorders are referred to as synucleinopathies [4-7]. These observations from human studies and related insights from multiple vertebrate and invertebrate animal models of *SNCA over*-expression (reviewed in: [8]) demonstrate that both wild-type and mutant forms of aSyn can induce neurodegeneration [9-13].

Given that aSyn inclusions are a pre-requisite feature of synucleinopathies, the processing of aSyn has been examined extensively in both *ex vivo *and *in vivo *models. These investigations have focused either on post-translational modifications of aSyn [14] or on mechanisms of degradation. Initially, a key role had been postulated for the ubiquitin proteasome pathway (UPP) in the degradation of aSyn, because mutations in two UPP-related genes, *Parkin *and *UchL-1 *have been shown to influence PD risk [15-18] and because molecular, cellular and animal studies linked these genes to UPP-dependent processing of aSyn [19-21]. However, growing evidence has indicated that the lysosome, as well as the proteasome, can mediate degradation of aSyn [22,23]. In general, proteins are sequestered within lysosomes by one of three known methods, *i.e.*, macroautophagy, microautophagy, and chaperone-mediated autophagy (CMA) (reviewed in [24]). Of these, it appears that aSyn can be a substrate for both macroautophagy and CMA [25-28].

Regardless of the exact autophagic pathway by which aSyn enters the lysosome, it is assumed that it undergoes rapid degradation by a proteolytic enzyme (or enzyme complex, referred to as 'synucleinase/s'). Cathepsins are lysosomal proteases whose enzymatic activity is conferred by critical residues, *e.g.*, serine, cysteine or aspartic acid. Cathepsin D (CathD) is a major lysosomal aspartyl protease composed of two disulfide-linked polypeptide chains, both produced from a single protein precursor [29]. Interestingly, CathD deficiency and its enzymatic inactivation in either humans, sheep, dogs or mice results in an early-onset, progressive and ultimately fatal neurodegeneration, which has been classified as one of several 'neuronal ceroid lipofuscinoses' (NCL) [30-34]. *In vitro *experiments indicated that the treatment of recombinant aSyn with CathD resulted in partial aSyn proteolysis [35]. Recently, Sevlever *et al*. confirmed a proteolytic effect of CathD in in vitro digestion studies and extended their work to lysosomal fractions using human neuroblastoma cells over-expressing *SNCA *cDNA [36].

Here, we first examined the ability of CathD to regulate both wild-type and mutant aSyn in a dopaminergic cell culture system, and then examined the brains of several CathD-deficient mammals with NCL for evidence of misprocessing of endogenous aSyn. Finally, we tested the effects of *ctsd *expression on neuronal aSyn toxicity in a well-described *Drosophila *model of synucleinopathy. Our data indicate that ectopically expressed CathD enhances degradation of wild-type and mutant aSyn proteins, and that absence or deficiency of CathD promotes aSyn aggregation and toxicity *in vivo*.

## Methods

### Plasmid constructs and establishment of MES-h*SNCA *cell model

The human *SNCA *gene contained in the pcDNA3.1 vector was a kind gift of Prof. P.T. Lansbury. The rat *SNCA *gene was cloned out of a rat brain cDNA library using the primers GATATCGCCACCATGGATGTGTTCATGAAAGGACTT (forward) and CAAGACTATGAGCCTGAAGCCTCTTCTAGA (reverse), and inserted into the pcDNA3.1 vector (Invitrogen) using the EcoRV and Xba1 restriction sites. pCMV-XL5 vector with and without the human *CTSD *gene was purchased from Origene's TrueClone collection. All constructs were isolated from DH5alpha E. coli using Qiagen's Maxiprep kit. To create the Ala30Pro, Glu46Lys, Ala53Thr, Ser129Ala, Ser129Asp, and Asp98Ala/Gln99Ala mutants of human *SNCA*, site directed mutagenesis was performed using Stratagene's QuikChange kit. All constructs were sequence verified prior to use.

The dopaminergic rodent mesencephalic cell culture system (MES 23.5 cells) was a gift of S Appel, Baylor College of Medicine. Cells seeded at a density of 6 million per 10 cm dish onto poly-D-Lysine coated plates (BD Falcon), were transiently transfected using Lipofectamine 2000 (Invitrogen Corp). Cells were routinely transfected with 0.5 μg (per 10 cm dish) of *SNCA *plasmid (either human wt, rat wt, or human mutant) and with 0–5 μg (per 10 cm dish) of wt *CTSD *plasmid. The total amount of DNA was normalized to 5.5 μg (per 10 cm dish) using empty pCMV-XL5 vector. 24 hours later, cells were washed with Tris-buffered saline and lysed in 140 mM NaCl, 50 mM Tris-HCl, pH 8.0, 1 mM EDTA, 0.5% Triton-X100, and 1× protease inhibitors, as described [37]. Lysates were centrifuged at 100,000 g for 30 min at 4°C; the top 2/3 of supernatants were removed and frozen in siliconized tubes at -80°C. The activity of LDH (lactate dehydrogenase) in the conditioned medium of transfected MES23.5 cells was routinely assayed; cellular metabolism was measured by the conversion of MTT [(3-(4,5-Dimethylthiazol-2-yl)-2,5-diphenyltetrazolium bromide) to formazan, as described [38]. For these standard toxicity assays, a positive control leading to 100% cell lysis (0.1% Triton-X 100 treatment) was run in parallel.

### Antibodies, Western blotting and ELISA

The rabbit polyclonal 7071AP, hSA-2 and mSA-1 antibodies (Abs) were raised and affinity-purified at Open Biosystems, Inc. () against recombinant, full-length aSyn, which had been HPLC- and mass spectrometry-characterized and subjected to amino acid composition and protein concentration analyses [39]. Cell lysates were analysed by SDS-PAGE as described [40]. The monoclonal aSyn antibody, Syn-1, was purchased from BD Clontech, the monoclonal anti-Ser129Phos antibody was from Dako, and the goat polyclonal anti-CathD antibody was from Santa Cruz.

Lysates were analysed for aSyn content by a 384-well quantitative sandwich ELISA (enzyme-linked immune-adsorbant assay), as described in detail elsewhere [39,40], using hSA-2 Ab for coating and biotinylated Syn-1 Ab as the readout Ab. Color development was carried out by using Fast-p-Nitrophenyl Phosphate (Sigma) and monitored kinetically at OD 405 nm every 5 min for up to 60 min. For serial dilutions of cell lysates on the ELISA plate, blocking buffer containing 0.5% lysate from vector-transfected cells was used as diluent; the same was used to create the corresponding standard curve of recombinant human aSyn (Additional file 1, Fig. S1A). Saturation kinetics were examined for identification of time point(s) where standards and sample dilutions were in the log phase. When analyzing cell lysates by ELISA [hSA2/Syn1-B], we recorded concentrations in MES-h*SNCA *cells that showed the expected parallelism after serial dilution (e.g., the 1:2000 dilution gave a value approximately twice that of the 1:4000 dilution; Additional file 1, Fig. S1B), that were *SNCA *cDNA dose-dependent (Additional file 1, Fig. S1C), and that permitted the precise calculation of the total aSyn protein concentration (pg per total protein in μg) expressed in living cells.

### Immunohistochemistry and histochemistry

*Ctsd *-/- mice produced by Saftig and colleagues [41] were maintained on a mixed C57BL6J strain background in the animal facility of the Helsinki University, Biomedicum, where food and water were available *ad libitum *and the light/dark cycle was 12/12 hours. The study protocol was approved by the Ethical Committee of Helsinki University. Mice were decapitated at the age of 24 days and brains were collected. For immunohistochemistry, one hemisphere was immersion fixed in 4 per cent neutral buffered paraformaldehyde, dehydrated and embedded in paraffin, while the other hemisphere was frozen for biochemistry.

Archival paraffin-embedded brain specimens of sheep with congenital ovine NCL (due to a homozygous missense mutation in the *CTSD *gene) and of three infants with congenital NCL (due to a homozygous duplication event in *CTSD *and an unknown gene defect leading to absence of CathD in tissue) were analysed by immunohistochemistry, as described [30,31]. Four μm-thin paraffin sections were dewaxed in xylene and descending alcohol series. Endogenous peroxidase activity was blocked by incubation in methanol containing 1.6% hydrogen peroxide, 30 min at RT. Sections were then rinsed 3 × with PBS and blocked with 15% normal serum, 30 min at RT, before O/N incubation at 4°C with primary antibody. Sections were washed 3 × 5 min with PBS and incubated with biotinylated secondary antibody and avidin coupled peroxidase for 30 min RT (mouse Vectastain Elite ABC kit, Vector laboratories, Peterborough, UK), and stained with aminoethyl carbazole as substrate. Sections were counterstained with hematoxylin. Immunohistochemistry of ovine brain sections was carried out as described in detail elsewhere [37,42].

The *SNCA*-expressing and the CathD-deficient *Drosophila *lines have been described previously [9,43]. These lines were crossed with each other to obtain flies expressing human aSyn wild-type (WT) in the absence of CathD. *Ctsd *genotypes were verified using a PCR-amplification of the fly *ctsd *locus on chromosome 2. Flies expressing either human aSyn WT in wt background and *ctsd*-null flies not expressing aSyn were used as controls. Flies were fixed in 4 per cent buffered paraformaldehyde, dehydrated and embedded in paraffin. Four mm-thin sections were stained with haematoxylin-eosin.

### Mouse brain homogenization and fractionation

For initial examination of the central nervous system, frozen brains (without cerebellum and brain stem) from 24-day old wt and *ctsd*-/- mice were homogenized in 40 mM Tris-Cl, pH 7.4 containing protease inhibitor cocktail (Complete, Amersham) and centrifuged at 1,500 g for 10 min at 4°C. These supernatants were used for Western analyses and called Tris-extracts. For serial extraction experiments, frozen brains (including the brain stem and cerebellum) from 24-day old wt and *ctsd*-/- mice were homogenized in lysis buffer (STEN LB, 50 mM Tris-HCl, pH 7.5, 140 mM NaCl, 2 mM EDTA, protease inhibitor cocktail, and 0.4 per cent NP-40), as described [37]. Homogenates were centrifuged at 100,000 g for 45 min at 4°C to collect the NP-40 supernatant. The pellet was suspended in STEN LB containing 1% SDS, followed by centrifugation at 100,000 g for 45 min at 12°C to collect the SDS supernatant; the remaining SDS pellet was suspended in 600 μl of STEN LB containing 1% formic acid (FA) and left overnight on a shaker at 4°C.

## Results

### Ectopic Cathepsin D effectively degrades wild-type and mutant α-synuclein in dopaminergic cells

**Figure 1:**
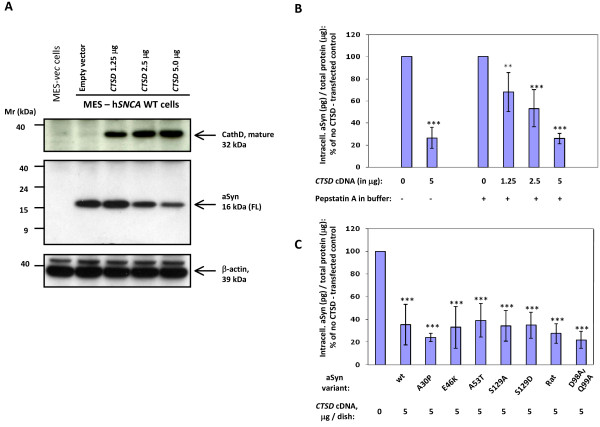
**Over-expression of Cathepsin D lowers α-synuclein in dopaminergic cells**. **(A) **Western blot analyses under reducing conditions of MES23.5 cells [38] expressing untagged, wild-type human *SNCA *(hSNCA WT) or vector (vec) plasmid DNA following transfection with increasing amounts of *CTSD *cDNA. Lysates collected 24 hours after transfection show an inverse decrease in the level of full-length (FL) α-synuclein (aSyn), as shown with polyclonal antibodies (Ab), 7071AP [46], hSA-2 [39,40], and monoclonal Ab Syn-1 (not shown); anti-β-actin was used as loading control. Note, no low molecular fragments of aSyn are detected under these conditions. **(B) **ELISA [hSA2/Syn1-B] and statistical analyses of MES-aSyn cells transfected with increasing amounts of *CTSD *cDNA, demonstrating the quantitation of the aSyn-lowering effect by ectopic Cathepsin D (**, P < 0.01; ***, P < 0.001). Note, the addition of Pepstatin A to the cell lysis buffer did not change the observed reduction of the intracellular aSyn concentration (up to < 80 per cent). **(C) **ELISA [hSA2/Syn1-B] and statistical analyses of MES23.5 cells demonstrating the quantitation of ectopic Cathepsin D expression (5 μg per dish) on mutant aSyn proteins and wild-type rat aSyn (see text for details). Data in (B) and (C) are expressed as mean [± standard deviation] of the intracellular concentration of aSyn (in pg/per μg total protein) in MES23.5 cell lysates from each dish and graphically displayed in per cent numbers of vector control without ectopic *CTSD *expression. Graphs reflect six independent experiments.

To investigate whether CathD has any effect on aSyn turnover, we utilized a rodent cell line of mesencephalic origin, MES23.5, which constitutively synthesizes dopamine [38,44,45]. At baseline, MES23.5 cells show very low levels of endogenous *ctsd *and *snca *gene expression (Fig. 1A). Therefore, we co-expressed untagged wild-type (wt) human *SNCA *cDNA with increasing amounts of untagged, wt human *CTSD *in these cells. After 24 hours of co-expression, we observed by Western blotting that as the levels of the mature, 32 kDa CathD protein increased, the signal intensity of the 16 kDa, full-length (FL) aSyn monomer decreased (Fig. 1A). Of note, this decrease in aSyn monomer was not accompanied by an increase in lower molecular weight fragments of aSyn, as examined by longer exposure of Western blots with four different antibodies that had previously shown reactivity with both FL aSyn and C-terminally truncated aSyn species *in vivo *(Syn-1, 7071AP; hSA-2; mSA-1; Additional file 2, Fig. S2) [39,46]. Similarly, none of these four antibodies detected any CathD-induced cellular accumulation of insoluble or higher molecular weight aSyn species. Furthermore, we confirmed by LDH and MTT assays that the observed effect of CathD on lowering of aSyn was not caused by a decrease in cellular integrity or metabolic activity. Importantly, co-expression of aSyn with plasmids encoding three other untagged, wild-type, human lysosomal enzymes (*i.e.*, cathepsin E, cathepsin L and sulphotransferase), which were expressed under the same promoter as *CTSD *and analyzed as above, did not lower the intracellular aSyn concentration (data not shown).

To quantify precisely the efficiency of CathD-induced reduction of aSyn in our cell culture model, we employed a sandwich-type enzyme-linked immunoabsorbent assay (ELISA [hSA2/Syn1-B pair]), that is both specific and sensitive for the quantification of cellular aSyn concentrations (Additional file 1, Fig. S1; [39,40]). As shown in Fig. 1B, we consistently recorded a significant, dose-dependent decrease in soluble aSyn with ectopic CathD expression, which peaked at 5 μg *CTSD *cDNA per 10 cm dish. Importantly, the effect of CathD on aSyn occurred in living cells, because the addition of the specific CathD inhibitor, pepstatin A, to our lysis buffer did not alter the outcome (Fig. 1B).

Heritable PD has been linked to distinct point mutations within the *SNCA *gene; furthermore, sustained phosphorylation of aSyn at residue Ser129 has been found in brain specimens of virtually all human synucleinopathies [14,47]. Therefore, we next examined the effect of human CathD on these aSyn variants by expressing degradation efficiency as a percentage of the signal obtained from each relevant mutant in the absence of CathD. As shown in Fig. 1C, CathD over-expression efficiently promoted the degradation of the three known PD-linked aSyn mutants, *i.e.*, Ala30Pro, Glu46Lys and Ala53Thr. Furthermore, CathD over-expression also efficiently degraded two other aSyn variants, which we engineered to abrogate and mimic phosphorylation at Ser129, *i.e.*, Ser129Ala and Ser129Asp, respectively. Similarly, exogenous CathD was able to lower the levels of full-length rat aSyn, using the same paradigm (Fig. 1C).

According to Cuervo and colleagues, residues Asp98 and Gln99 represent a putative motif by which aSyn is recognized by the hsc70 chaperone complex preceding chaperone-mediated autophagy and degradation [28]. To investigate the importance of this motif for CathD in lowering aSyn *ex vivo*, MES23.5 cells were transfected with an aSyn variant where these two critical residues were changed to Alanine (DQ/AA-aSyn); CathD expression reduced DQ/AA aSyn as efficiently as wt aSyn in these MES23.5 cells when analyzed 24 hours after transfection (Fig. 1C).

### Soluble α-synuclein levels are reduced in Cathepsin D-deficient mouse brain

**Figure 2:**
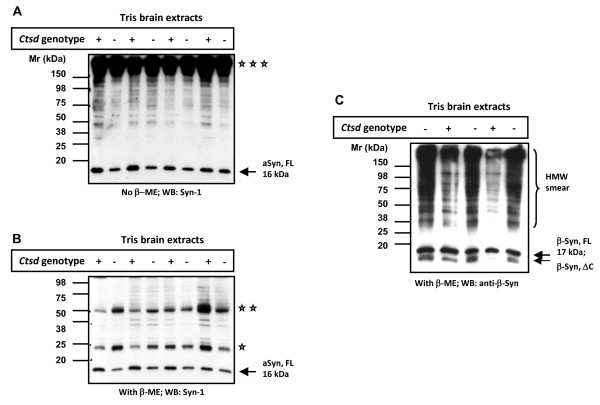
**Mice lacking *ctsd *gene expression show reduced levels of endogenous α-synuclein in Tris-soluble brain extracts**. Western blot analyses of Tris brain extracts from 24-day old, wild-type (+) and *ctsd*-null mice (-) carried out under non-reducing **(A) **and reducing **(B) **conditions. Probing with monoclonal aSyn Ab, Syn-1 (and rabbit anti-mouse IgG secondary Ab) demonstrates a subtle reduction in the full-length, monomeric aSyn protein (aSyn, FL) at the 16 kDa position in *ctsd*-null mice. Representative examples of 24 mouse brains are shown (n = 4 per genetic group under non-reducing conditions; n = 16 per genotype under reducing conditions). The same blots reveal a broad, high molecular weight (HMW) band above 150 kDa (triple asterisks), and reactive bands at 50 kDa (double asterisks) and 25 (asterisk) kDa positions under non-reducing (A) and reducing (B) conditions, respectively. (**C) **Immunoblotting with anti-β-synuclein Ab under reducing conditions reveals no difference at the 17 kDa monomeric (β-Syn, FL) protein position, but indicates elevation of lower molecular weight fragment(s) at ~13 kDa (β-Syn, ΔC) and a HMW-immunoreactive smear in cathD-deficient mice. (Note, no additional anti-β-synuclein Ab is available for corroboration).

To determine the role of CathD on endogenous aSyn metabolism *in vivo*, we utilized a well characterized *ctsd *knock-out (*ctsd*-/-) mouse model. These mice carry two null alleles of murine *ctsd *and develop a fatal NCL leading to premature death at day 26 ± 1 [33,41]. We first examined aSyn levels in the brain of *ctsd*-/- mice and their wild-type (wt) littermates by SDS/PAGE and Western blotting under both non-reducing (Fig. 2A) and reducing conditions (Fig. 2B). We found that the 16 kDa aSyn monomer was slightly but consistently less abundant in Tris-extracts of *ctsd*-/- brain (without cerebellum and brain stem) when compared with littermate controls (n = 4 per group for non-reducing conditions; n = 16 per group for reducing conditions). In these extracts, we found no evidence of truncated aSyn species by Western blotting (Fig. 2A, B; and data not shown). In parallel, the amount of monomeric, full-length β-synuclein protein (17 kDa) appeared unchanged in *ctsd*-/- versus wt control mouse brains (Fig. 2C), although higher molecular weight species (and possibly truncated variants) appeared increased (Fig. 2C; note, the latter findings could not be corroborated with another Ab due to lack of availability). In addition to the somewhat unexpected reduction of the 16 kDa aSyn protein in *ctsd*-null brains, we observed that the mouse monoclonal aSyn Ab, Syn-1, also visualized bands that migrated at molecular weights of 25 kDa, 50 kDa (reducing conditions; Fig. 2B) and > 150 kDa positions (non-reducing, Fig. 2A). These bands were interpreted as endogenous immunoglobulin chains, as they were also detected by anti-mouse secondary Ab alone (not shown).

**Figure 3:**
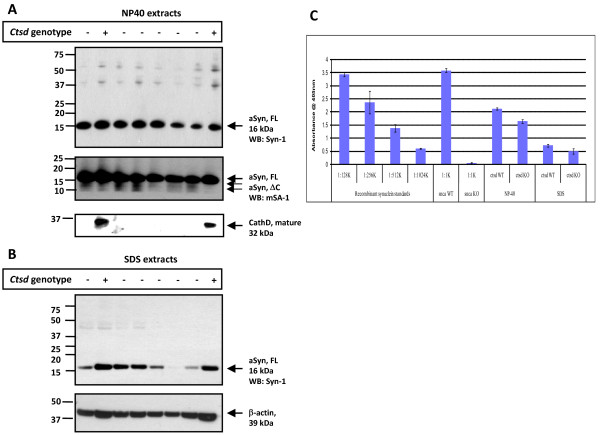
**CathD-deficient mice show reduced α-synuclein levels in detergent soluble brain extracts**. Western blot analyses of fractionated whole brain homogenates from 24-day old, wild-type (+) and *ctsd*-/- mice (-) carried out under reducing conditions showing NP-40 **(A) **and SDS extracts **(B)**. Immunoblots were probed with anti-aSyn Abs (Syn-1; mSA-1), anti-β-actin and anti-CathD Abs. Primary Ab detection was carried out with sheep anti-mouse and donkey anti-rabbit Ab, respectively. Under these immunoblotting conditions, variable amounts of the 16 kDa aSyn monomer (aSyn, FL) and traces of 12.5 kDa and 10 kDa (aSyn, ΔC) aSyn-reactive proteins are detectable; no reproducible > 150, 50 and 25 kDa aSyn-positive bands are seen.**(C) **ELISA [hSA2/Syn1-B] quantification of NP-40 and SDS extracts from *ctsd *wild-type (WT) and mutant *ctsd*-/- (KO) mouse brains and of *snca *WT and *snca*-/- (KO) lysates serving as positive and negative controls. *Ctsd*-genotyped brain extracts were loaded in triplicates at three different dilutions each (1:500 to 1:2,000) onto a 384-well plate and read at 405 nm, as described [39,40]. Note, the absence of OD405 nm signals in *snca *KO mouse tissue. Bar graphs represent the mean group values (± SD) of mice that were individually analyzed (numbers as in A-C). The concentrations of total aSyn (in ng/μl) present in NP-40 extracts measured: *ctsd *WT, 1.34 ± 0.25; *ctsd *KO, 1.09 ± 0.26; and in the SDS fractions: *ctsd *WT, 0.60 ± 0.25; and *ctsd *KO, 0.48 ± 0.28.

In order to further substantiate and expand these observations, we carried out a serial fractionation protocol of whole mouse brain (including cerebellum and brain stem) using an initial NP-40 detergent extraction step followed by a SDS extraction step of the remaining NP-40 pellets. There, we blinded the operator regarding the genotype, analyzed fractions by both immunoblotting with multiple Abs (Fig. 3A, 3B) and by sandwich ELISA (Fig. 3C), and then confirmed the genotype by probing for the presence (or absence) of CathD in all specimens (Fig. 3A). In agreement with the results obtained in Tris extracts, our serial fractionation and ELISA quantification demonstrated a reduction – rather than an increase – in the levels of soluble aSyn in *ctsd*-/- mouse brains (n = 8) when compared with their WT (n = 4) littermates; the reduction measured 18.6 and 20.0 per cent in NP-40 and SDS fractions, respectively (Fig. 3C). In addition, using densitometry we quantified the low molecular weight species of aSyn (migrating at 12.5 and 10 kDa positions) that were detected in NP-40 extracts by mSA-1 (Fig. 3A; Additional file 2, Fig. S2), but we found no apparent difference between the two genotypes (not shown).

### Insoluble α-synuclein oligomers are elevated in Cathepsin D-deficient mouse brain

**Figure 4:**
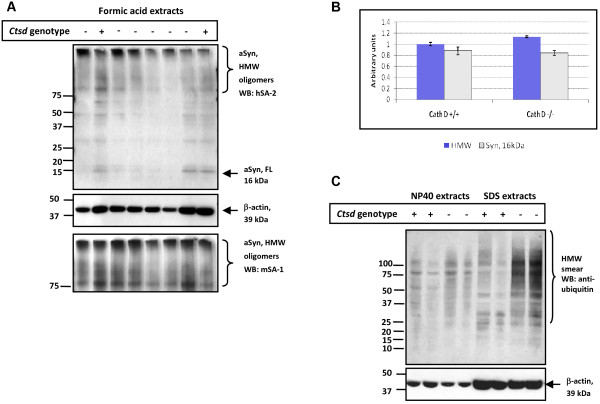
**CathD-deficient brains show increased HMW α-synuclein-positive oligomers in formic acid extracts**. **(A) **Western blot analyses carried out under reducing conditions of formic acid extracts obtained from previously generated SDS pellets of wild-type (+) and *ctsd*-/- (-) mouse brains (see Fig. 3 above). Immunoblots were probed with high sensitivity, polyclonal aSyn Abs, hSA-2 and mSA-1, and anti-β-actin Ab, as indicated. Under these conditions, HMW aSyn-positive smears and trace amounts of monomeric, 16 kDa aSyn (aSyn, FL) are detectable in these formic acid extracts.**(B) **Quantification of aSyn-reactive species by densitometry examining the ratio of HMW smears (blue bars) and 16 kDa monomer (empty bars) versus the signal of β-actin-positive bands. Note, an ~15 per cent increase in HMW oligomers of aSyn is detected in formic acid extracts of *ctsd*-/- brain by independent analysis of both mSA-1 (B) and hSA-2 immunoblots. **(C) **NP-40 and SDS extracts probed with anti-ubiquitin consistently reveal HMW immunoreactive smears in SDS brain extracts of *ctsd*-/- mice. Representative examples of twelve mouse brains are shown (n = 4, *ctsd*+/+; n = 8, *ctsd*-/-).

To complete the serial extraction protocol, we next searched for the presence of insoluble aSyn species in the formic acid extracts of SDS pellets from the *ctsd*-/- mouse brains shown in Fig. 3. There, we specifically searched for HMW aggregates of aSyn (referred to as oligomers) employing our most sensitive Abs (Fig. 4A). Independent Western blotting with hSA-2 and mSA-1 (Fig. 4A) and subsequent densitometry of the ratio, aSyn to actin, revealed more insoluble, HMW oligomers in *ctsd*-/- than in WT mouse brain (Fig. 4B). Of note, these oligomers migrated predominantly at the interface of the stacking gel and running gel; their difference between the two genotypes was estimated to be ~15 per cent, as detected by both Abs (Fig. 4A and 4B; and data not shown). Intriguingly, these formic acid extracted aSyn oligomers evidently did not co-localize with the HMW ubiquitin positive smears, as the latter were predominantly found in SDS extracts (Fig 4C).

### Cathepsin D deficiency promotes intracellular α-synuclein accumulation in mouse brain

**Figure 5:**
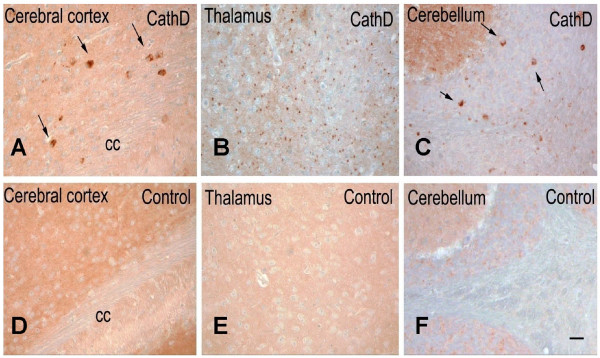
**Mice lacking *ctsd *gene expression exhibit intracellular α-synuclein accumulation in several regions of the brain**. Serial, paraffin-embedded sections from 24-day old cathepsin D knock-out mice (CathD) **(A-C) **and age-matched, wild-type control **(D-F) **mice (n = 5) were probed by immunohistochemistry with affinity-purified, polyclonal aSyn Ab, hSA-2. **(A) **In the frontal cortex of cathepsin D knock-out mice, scattered neurons with prominent intracellular aSyn-reactivity are detectable (arrows). **(B) **In the thalamus of CathD-deficient mice, the neuropil staining is reduced when compared with control animals, and small grain-like aggregates of aSyn are visible. **(C) **In the cerebellum of CathD deficient mice, abnormal aSyn-positive accumulations of varying size can be observed in the granular cell layer and deep nuclei; those that appear cytoplasmic are identified by arrows. **(D-F) **As expected, aSyn-positive aggregates are not found in age-matched, wild-type mice processed in parallel. CC denotes corpus callosum. Scale bar, 25 μm.

Our biochemical data showed a reduction of detergent soluble aSyn and an increase of formic-acid soluble aSyn species in *ctsd*-/- mouse brains. In human PD, DLB and MSA brain, the formic acid-soluble or urea-soluble fractions contain HMW aSyn aggregates. Therefore, we next explored whether any aSyn aggregates were also detectable in brains of 24-day old *ctsd*-/- mice by immunohistochemistry (Fig. 5). To avoid any cross-reactivity of the secondary anti-mouse Ab with endogenous immunoglobulins, we employed our rabbit polyclonal, affinity-purified anti-aSyn Abs (mSA-1 and hSA-2), whose binding specificity for mammalian aSyn was confirmed using *snca*-null tissue (Additional file 2, Fig. S2) [39,40,48,49]. As shown in Fig. 5, we recorded prominent aSyn staining in the neuropil of the cerebral cortex, thalamus and cerebellum of control mice, as expected from its known presynaptic localization (Fig. 5D to 5F). In contrast, brain sections of *ctsd*-/- mice, processed in parallel, showed a less prominently stained neuropil, particularly in the thalamus (Fig. 5A, B). Intriguingly, in these CathD-deficient mice, we also detected aSyn-positive aggregates within select neurons of the deep cortical laminae, superior colliculi, the subiculum, as well as in the thalamus, deep cerebellar nuclei, and even within white matter tracts (Fig. 5A–C). Intra-neuronal cytoplasmic accumulations appeared large, while those within the thalamus were relatively small, thereby suggesting their localization within neurites. We concluded from these collective mouse data that: one, the distribution and processing of endogenous aSyn is altered in *ctsd*-/- mice; and two, murine CathD deficiency promotes an early-onset NCL phenotype that features the accumulation of a small pool of aSyn within an insoluble brain compartment.

### Mutant Cathepsin D expression alters α-synuclein immunohistochemistry in sheep brain

**Figure 6:**
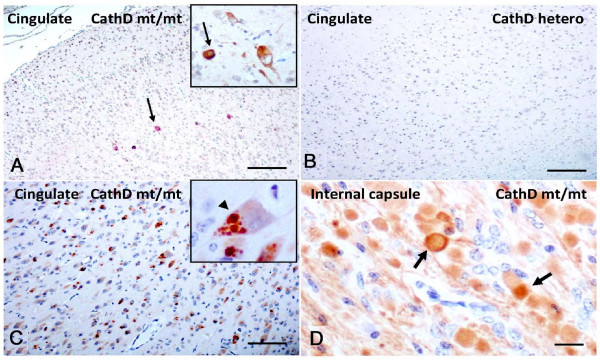
**Sheep expressing a homozygous mutant of Cathepsin D exhibit accumulation of phosphorylated α-synuclein in cingulate cortex**. **(A) **Select cortical neurons (arrow) of cingulate gyrus in homozygous mutants (CathD mt/mt) show cytoplasmic immunostaining with Ser129-phosphorylated aSyn Ab. **(B) **Absence of Ser129-phosphorylated aSyn-reactivity in the cingulate gyrus of age-matched, heterozygous *CTSD *(CathD hetero) sheep brain. **(C) **Anti-ubiquitin antibody staining reveals extensive, often multiple, intra-cellular inclusion formation in neurons (arowhead) of affected sheep throughout the cortex including cingulate cortex. **(D) **Immunostaining for aSyn with polyclonal Ab, hSA-2, reveals abundant aSyn deposits and axonal swelling (arrows) in the internal capsule of homozygous mutant sheep but not wild-type (not shown) or heterozygous sheep (not shown). Scale bars, 100 μm in A, B, C, and 15 μm in D.

To validate these results in a second mammal, we carried out immunohistochemical studies in a sheep colony affected by congenital NCL (Fig. 6) [31]. This phenotype is caused by a homozygous missense mutation in the ovine *CTSD *gene (Gln934Ala), which renders the enzyme proteolytically inactive despite a 5- to 10-fold over-abundance of the mutant protein in the forebrain [50]. In surveys of sagittal hemibrain sections with our panel of mono- and polyclonal Abs to mammalian aSyn, overall staining of the neuropil appeared relatively normal in affected sheep (Fig. 6A; n = 3 for NCL sheep; n = 1 for heterozygous sheep; n = 2 for wt control sheep). However, staining with our polyclonal Abs for ovine aSyn demonstrated widespread areas of elongated axonal swelling in deep white matter tracts in affected sheep (Fig. 6D) and revealed isolated aSyn aggregates in the thalamus, similar to the mice (not shown). As expected, no axonal abnormalities or aggregates were seen in heterozygous (Fig. 6B) and wt animals (not shown). Intriguingly, when we probed the sections with a well characterized monoclonal Ab to phosphoSer129-aSyn, we observed a small number of neurons in the cingulate cortex that showed intense intracellular labeling in homozygous *CTSD *mutant sheep (Fig. 6A), but not in heterozygous (Fig. 6B) and wt sheep. Of note, the axonopathy detected by the polyclonal Abs to aSyn was not matched by anti-phosphoSer129-aSyn reactivity. The changes in aSyn reactivity described above appeared specific, as staining of CathD-deficient sheep brain sections with Abs to Alzheimer's-linked phosphorylated tau, and TDP-43 did not reveal any obvious abnormalities (not shown). In contrast, development of sections with a monoclonal anti-ubiquitin Ab revealed extensive inclusion formation that was present in NCL sheep brain throughout the cortex (Fig. 6C), including the cingulate cortex, but was absent in heterozygote and wt animals.

### Cathepsin D deficiency promotes α-synuclein accumulation in human newborns

**Figure 7:**
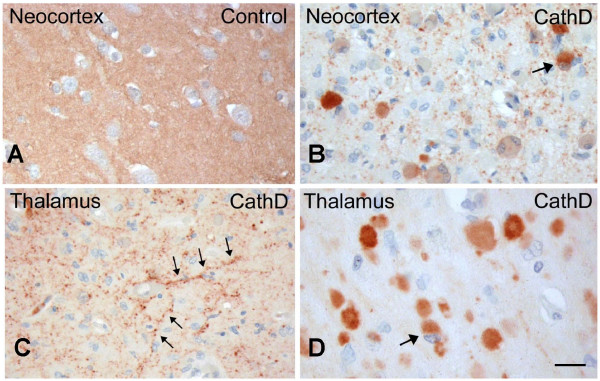
**Human neuronal ceroid lipofuscinosis due to Cathepsin D deficiency leads to intracellular accumulation of α-synuclein**. **(A) **Paraffin-embedded brain sections of a neurologically normal infant (Control) stained with polyclonal aSyn Ab, hSA-2, show strong reactivity of the neuropil in the neocortex. **(B) **Immunohistochemistry of brain specimens from CathD-deficient infants (CathD; n = 3), carried out in parallel, demonstrates marked reduction of neuropil staining. These sections also display scattered, cytoplasmic aSyn aggregates within neurons (identified by arrow). **(C) **Sections of the thalamus from the same individual as in B also reveal neuritic pathology (arrows), as well as **(D) **intracellular accumulation of aSyn, sometimes seen as a juxtanuclear inclusions (arrow). Scale bar, 15 μm.

Next, we analyzed serial sections of paraffin-embedded, *post mortem *human brains from three infants diagnosed with congenital NCL (Fig. 7). One of the three patients carried a homozygous duplication (c.764 dupA) in exon 6 of the *CTSD *gene, thereby creating a premature stop codon and loss of CathD expression; another patient was his older sibling, who died of the same disease phenotype and thus, most probably, carried the same mutation, while the third, unrelated patient with congenital NCL had no detectable CathD protein present in her tissues although she remains genetically unidentified [30]. The brains of these three children revealed extreme atrophy with massive neuronal loss throughout the cortex, accompanied by generalized glial activation [30,51]. Using the same Ab panel employed above, we observed markedly reduced aSyn staining in the cortical neuropil of affected infants (Fig. 7B) compared to control brain (Fig. 7A), similar to the changes seen in *ctsd*-/- mutant mouse brain above. In addition, we identified strongly aSyn-reactive neurons scattered throughout the neocortex (Fig. 7B). These neurons carried cytoplasmic aSyn accumulations that appeared granular and were visible throughout the soma (Fig. 7B). Prominent 'synucleinopathy' was also observed in deep grey matter structures, thalamus (Fig. 7C, D) and basal ganglia of infants with congenital NCL. There, the aggregates varied greatly in size, with some appearing to localize to proximal axons (Fig. 7C), and some to the cell soma (Fig. 7D). Anti-hSA2-positive deposits could also be seen within white matter tracts, thus confirming their axonal localization. Within the cerebellum, aSyn deposits were observed in the granular cell layer and deep white matter. Of note, these inclusions were negative for Ser129-phosphorylation-specific aSyn reactivity, and for ubiquitin (not shown). As expected, no such aSyn abnormalities were seen in the control brain of a neurologically normal infant processed in parallel (Fig. 7A).

### Neurotoxicity of human α-synuclein in Drosophila is enhanced in ctsd-mutant flies

**Figure 8:**
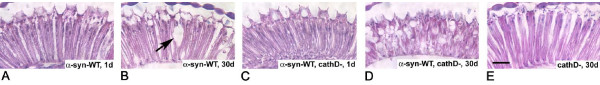
**Cathepsin D deficiency enhances α-synuclein toxicity in the retina of fly brain**. **(A) **Normal retina in one-day old, human *SNCA *cDNA-transgenic flies (α-Syn-WT; Feany and Bender, 2000). **(B) **At day 30 these flies show mild degeneration; arrow indicates vacuole. **(C) **Normal morphology of retina at day 1 in flies expressing human *SNCA *cDNA in the absence of endogenous fly cathD (α-Syn-WT, cathD-). **(D) **Marked degeneration of retinal neurons occurs by day 30 in flies ectopically expressing human *SNCA *cDNA in the absence of fly cathD. Note the appearance of numerous vacuoles, and the thinning and loss of retinal architecture. **(E) **Deletion of fly *ctsd *in the absence of aSyn expression (cathD-)does not cause retinal degeneration at day 30. Scale bar, 20 μm.

Over-expression of aSyn has been shown to be neurotoxic in a variety of cellular and animal models. In order to study the effect of endogenous CathD on aSyn-induced neurotoxicity, we generated flies expressing human aSyn in a *ctsd*-null background. In agreement with our previous results [9], the retina was normal in one-day-old transgenic flies over-expressing wt human aSyn (Fig. 8A), but showed mild, vacuole-carrying changes by day 30 (Fig. 8B). Flies expressing human aSyn in the absence of CathD also had normal retinas at day one (Fig. 8C), but developed pronounced retinal degeneration within 30 days; these progressive changes included the formation of numerous vacuoles, thinning of the retina and loss of its architecture (Fig. 8D). Importantly, fly CathD deficiency by itself (without aSyn expression) did not cause this neurodegeneration phenotype of the retina at day 30 (Fig. 8E; [43]). These results indicated that neurotoxicity of aSyn is enhanced in the absence of endogenous CathD expression.

## Discussion

This report summarizes complementary investigations into the role of a key lysosomal enzyme, CathD, in the processing of aSyn. The *in vivo *results from three mammalian and one insect species described here provide additional insights into the pathogenetic effects of CathD deficiency, and identify in CathD a candidate for the much sought after 'synucleinase' activity *in vivo*.

Our initial set of experiments focused on a dopaminergic neuroblastoma cell culture model over-expressing aSyn together with CathD (Fig. 1). *CTSD *expression reduced aSyn concentrations in a manner that was dependent on the level of newly synthesized CathD (Fig. 1A and 1B). This effect could not be explained by a loss of cell viability, as the integrity of our culture model was unchanged from untransfected and vector-transfected cells in two standard viability assays. Similarly, the effect of *CTSD *cDNA could not be explained by a competition effect with the *SNCA *plasmid for the protein synthesis machinery, because co-expression of aSyn with Cathepsin L (CathL) or Cathepsin E (CathE) under the same conditions did not cause a reduction of aSyn concentrations (data not shown).

The simplest explanation for the effects of *CTSD *expression on aSyn is that CathD-mediated degradation of aSyn within the lysosome was enhanced (see alternative explanations below). For this explanation to be valid, aSyn must have entered the lysosome by either macroautophagy or CMA, or both, given that CathD is thought to be active only at low pH. Several investigators have shown that the Asp98 and Gln99 residues of aSyn facilitate recognition by the Hsc70 chaperone and subsequent binding of the aSyn/Hsc70 complex to the Lamp2a receptor during CMA [26,28,52]. According to Cuervo *et al.*, Ala30Pro and Ala53Thr mutants of aSyn are able to bind to isolated lysosomal membranes but unable to translocate into the lysosomal lumen for degradation [28]. The fact that in our cell culture system, the D98A/Q99A double mutant of aSyn, as well as the Ala30Pro, Glu46Lys and Ala53Thr mutants of aSyn were degradable by exogenous CathD (Fig. 1C) suggests that, at the collection time point of 24 hours, the cells did not entirely rely on CMA as a means of transporting aSyn into lysosomes, and that, rather, macroautophagy could be equally, or even more important than CMA [26,52]. Likewise, changing the phosphorylation state of human aSyn at Ser129 did not alter the proteolytic synucleinase activity exhibited by CathD in our MES23.5 cell model. Of note, only select neurons in sheep cingulate cortex deficient for lysosomal CathD activity (but not in mouse or human brain) revealed evidence for abnormal anti-Ser129-aSyn reactivity (Fig. 7 and data not shown). Although the observed species difference could be caused by a variety of factors, our cellular data argue that Ser129 phosphorylation of aSyn is not a universal pre-requisite for CathD-induced aSyn degradation.

Western blotting analyses of our MES23.5 cell lysates demonstrated that the CathD-induced reduction in aSyn occurred without the concomitant appearance of either low-molecular weight (LMW) or HMW species, as examined 24 hours after transfection. Of note, we had previously confirmed by Western blotting that our Abs are capable of recognizing truncated aSyn species, (*i.e.*, 10 to 12.5 kDa; see Additional file 2, Fig. S2) [46]. In particular, the appearance of LMW species of aSyn would have indicated incomplete proteolysis of aSyn, potentially at the C-terminus. Such LMW aSyn-ΔC species are prone to aggregate formation and have an enhancing effect on aSyn-mediated toxicity [46,53-55]. Previous studies by Dufty *et al. *[56] showed that calpain-mediated digestion of aSyn could induce the formation of aSyn-ΔC species and formation of HMW species. Recently, Sevlever *et al. *confirmed a proteolytic effect of both enzymes, calpain and CathD, in *in vitro *biochemical studies and extended their work to purified lysosomes from *SNCA*-expressing neuroblastoma cells [36].

In our *SNCA *and *CTSD *over-expression model, we observed a maximum effect of CathD-induced reduction of excess aSyn levels that plateaued at ≤ 80 per cent of vector-transfected cells (Fig. 1B). We concluded from these findings that a distinct pool of aSyn species (e.g., synaptic vesicle-associated) is not amenable to CathD-mediated aSyn degradation. To explore whether over-expression of wt *CTSD *also reduces the concentration of endogenous aSyn, we carried out additional Western blotting and ELISA experiments in rodent MES23.5 and human SY5Y neuroblastoma cells. In the background of normal lysosomal function and normal aSyn levels, we saw no consistent, further reduction of endogenous aSyn levels by ectopic CathD expression (not shown).

To examine the relevance of endogenous CathD expression on endogenous aSyn processing *in vivo*, we next examined brain tissue from homozygous *ctsd*-/- mice [41]. We detected a consistent decrease in soluble aSyn proteins in homozygous CathD deficient mouse brains *versus *those of wt animals using Western blotting and ELISA (Figs. 2 and 3). This result was verified by quantitative 2D gel electrophoresis, which showed a significant reduction of the 16 kDa aSyn spot in *ctsd*-/- mice (data not shown). Our quantitative ELISA showed a comparable decrease in the amount of soluble aSyn in NP-40 extracts (18.6 per cent) and in SDS-extracts (20.0 per cent) of *ctsd*-/- mice compared with controls (Fig. 3). This observation was in agreement with our immunohistochemistry results from the same mice that exhibited some reduction in aSyn staining in the neuropil of *ctsd*-/- mice, particularly within the thalamus (Fig. 5). Previously, we have confirmed that CathD deficient mice show a decrease in pre-synaptic markers in the ventral posterior nucleus of the thalamus, and a > 30 per cent loss of neurons in its corresponding target somatosensory cortex [34]. Because aSyn is a predominantly pre-synaptic protein, we surmised that the reduction of soluble aSyn concentration recorded in our *ctsd*-/- mice resulted – in part – from pre-synaptic abnormalities.

However, in parallel to the reduction of soluble aSyn throughout the neuropil, we detected aSyn accumulation-carrying neurons within many brain regions of 24-day old *ctsd*-/- mice. This finding was substantiated by our observation of increased levels of insoluble species of oligomeric aSyn in the formic acid extracts of *ctsd*-/- mice (15%; Fig. 4). (Of note, our current ELISA protocol is incompatible with the quantification of aSyn in the presence of formic acid). We concluded from these collective findings that the presence of murine CathD is important for the prevention of aSyn misprocessing and that its absence facilitates the formation of aSyn aggregation *in vivo*. In accordance, we speculate that the neuronal accumulation of murine aSyn in *ctsd*-/- mice may represent early precursor lesions to classical Lewy inclusions.

We next examined the role of mutant CathD protein expression on sheep aSyn levels (Fig. 6). Homozygous missense mutation in the ovine *CTSD *gene (Gln934Ala) essentially abolishes the enzyme's proteolytic activity (residual level, < 5 per cent), while heterozygotes retain 40 per cent activity and are phenotypically normal [50]. The affected newborn lambs are weak, tremble and are unable to stand. When sacrificed shortly after birth, their brains are smaller and display mild cortical thinning, demyelination, neuronal loss, reactive astrocytosis and macrophage infiltration, while visceral organs are spared. Classical NCL-type changes and widespread ubiquitin-positive inclusions within neurons were readily detected in the homozygous animals (Fig. 6). In contrast to the more extensive neuropil disruption and synucleinopathy seen in *ctsd*-/- mouse brain, we detected more select neuronal changes in homozygous CathD-mutant sheep, with abnormal anti-aSyn staining in the cingulate cortex and in the axons of deep white matter tracts. Intriguingly, synucleinopathy of the cingulate cortex is a hallmark of human dementia with Lewy bodies and advanced PD cases [6]. Unfortunately, no frozen material was available to determine whether insoluble, HMW oligomeric species of ovine aSyn can be found biochemically in the cingulate cortex.

Humans with congenital NCL are affected at birth and survive only for days. Two mutations in the human *CTSD *gene have been described to underlie a subset of congenital NCL cases [30,57]. Both mutations inactivate the CathD enzyme, in that the insertion mutation, c.764dupA, results in a premature stop-codon and rapid degradation of the protein, while the c.299C > T mutation leads to the production of an inactive but stable protein *in vivo*. The brains of these infants show extreme cortical atrophy, loss of neurons and myelin, as well as generalized gliosis [30,51,57]. In brain specimens of two affected siblings from a NCL pedigree linked to *CTSD *and of one unrelated patient with congenital NCL, anti-aSyn staining of the neuropil was strongly reduced throughout the brain (Fig. 7). Accumulation of aSyn was particularly pronounced in the thalamus and basal ganglia, where the neuronal soma appeared to be loaded with aSyn aggregates, thereby morphologically resembling Lewy bodies of the forebrain; however, in contrast to classical Lewy inclusions these lesions were negative for ubiquitin and phospho-Ser129-specific reactivity in the affected newborns (not shown). Throughout the brains from these three infants, smaller aSyn-positive grains appeared to localize to neurites (Fig. 7B and 7C). Therefore, although the degree of brain atrophy and neuronal loss was much more severe in human infants with CathD deficiency than in *ctsd*-/- mice, the aSyn-reactive histopathologies were very similar between the two species, possibly because of the complete loss of CathD protein expression in both.

Regarding the topographic distribution of aSyn accumulation within the three species of CathD-deficient mammals, it is interesting to note that the latter is markedly different from that of lipofuscin deposition in NCL diseases. These autofluorescent accumulations are generally widespread within cortical neurons in humans and sheep affected by CathD deficiency and in the thalamus of *ctsd*-/- mice, while the observed aSyn accumulations occurred predominantly in the thalamus and basal ganglia in humans, in the cingulate cortex of affected sheep, and in the cerebral cortex of *ctsd*-/- mice.

Finally, our experiments using the established *Drosophila *model of PD provided evidence for a modulatory effect of endogenous CathD expression on aSyn toxicity *in vivo *(Fig. 8). Deletion of the homologous CathD-encoding gene promoted toxicity of ectopically expressed human aSyn in the fly retina; these findings suggest that differences in the expression level of CathD (and by inference, of other synucleinase-conferring proteases) may lower the threshold for synucleinopathy in other brain regions and species, too.

Importantly, our data do not demonstrate a direct interaction between CathD as an enzyme and aSyn as its substrate. Although such a relationship was suggested by *in vitro *experiments in one study [35], purification of lysosomes from co-transfected MES23.5 cells and detailed imaging analyses will be required to further test this hypothesis. Intriguingly, aSyn transgenic mice show a decrease in aSyn inclusions when immunized with recombinant aSyn, and this decrease was associated with a concomitant increase in CathD levels as well as co-localization of CathD and aSyn [58]. Stefanis *et al. *[59] also noted co-localization of a pool of aSyn with CathD in their PC12 cell line-based experiments.

Indirect effects on aSyn accumulation by CathD deficiency are also possible. For example, *CTSD *gene expression could activate another cathepsin or non-cathepsin protease, and this second enzyme may process aSyn for degradation. In this regard, it is interesting to note that CathD possesses activity that is similar to pepsin, and that pepsin can proteolytically activate Cathepsin F (CathF; [60]); intriguingly, in our cell culture system, over-expression of CathF was able to reduce human aSyn levels, too, with an efficiency similar to that of CathD, whereas over-expression of CathE and CathL was ineffective (V.C., M.G.S., unpublished observations). It is noteworthy that mice deficient in CathF develop a late-onset neurological phenotype with gait disturbance, hind leg weakness and tremors, which is associated with brain and spinal cord pathology [61]; it will be interesting to discern whether these mice also display biochemical and/or immunohistochemical evidence of synucleinopathy.

Another possible mechanism for interaction between the *CTSD *and *SNCA *genes *in vivo *is a change in lysosomal constituency downstream of CathD protein expression. Perturbations in the activities of several lysosomal sphingolipid-metabolising enzymes have been documented in CathD-mutant sheep [31] and dogs [32], while *ctsd*-/- mice accumulate phospho- and glyco-sphingolipids, including gangliosides [62]. Since aSyn is a lipid- and ganglioside-binding protein [63-65], and since lipids can regulate the oligomerization of aSyn [45,66], it is plausible that dysregulated lipid metabolism is a mechanism by which CathD deficiency leads to aSyn misprocessing. CathD is also known to exhibit activities that are unrelated to its enzymatic function [67-69]. It is therefore possible that non-proteolytic effects of CathD are essential in aSyn processing, akin to its effects on the ras-MAPK pathway [70]. Our data on aSyn processing in the cell soma and neurites of mutant sheep brain, where CathD is over-expressed but enzymatically disabled, would rather argue against this theory. However, one cannot exclude with certainty that the homozygous mutation in CathD did not also compromise its non-enzymatic functions, although computational modeling did not predict the induction of a conformational change in the sheep mutant (J.T., data not shown).

Finally, our collective findings reported herein are in remarkable agreement with many (but not all) of the findings in the related report by Qiao and coworkers [71], who independently discovered CathD as a critical enzyme in the metabolism of neuronal aSyn using both *in vitro *and *in vivo *experiments. Of note, Qiao *et al. *examined offspring of the same CathD-deficient mouse model that we employed [41], and observed a reduction of truncated aSyn species in *ctsd*-null mice, which we did not detect in our protocol. In addition to possible technical and Ab-related differences between the two reports, the slightly younger age of our animals at the time of euthanasia (post-natal day 24 *versus *25) may also have contributed to this discrepancy.

## Conclusion

These findings add NCL disorders to the growing list of human lysosomal storage diseases that feature misprocessing of neuronal aSyn [72-74]. Our evidence for altered processing and accumulation of insoluble species of endogenous aSyn in three different mammals with CathD deficiency indicates that its enzyme activity plays an important role in aSyn metabolism. This concept is supported by the pathogenesis replicated in our invertebrate *Drosophila *model, where the absence of CathD markedly enhanced aSyn toxicity *in vivo*. Although we have not yet revealed the molecular underpinnings for this effect, we consider the finding of CathD as a facilitator of 'synucleinase' activity an important, first lead in target identification.

The current treatment approaches for PD and related disorders are symptomatic at best, are not yet driven by molecular clues, and are invariably of limited duration. To date, progress in cause-directed treatment of synucleinopathies has been prevented mostly by our limited knowledge of the normal regulators that control aSyn steady-state *in vivo*, from *SNCA *transcription to aSyn degradation. Further exploration of the mechanism by which CathD promotes synucleinase activity holds the promise for novel drug targets to ultimately treat PD (and related disorders) at one if its root causes.

## Competing interests

VC and MGS are listed as co-inventors of an application to the US Patent Office in 2007 related to the development of novel therapeutics for Parkinson disease. Otherwise, the authors have no competing interests.

## Authors' contributions

VC and ML designed and conducted experiments; MGS and JT oversaw the project and together with VC wrote the manuscript; JN, AP, ES, PK and HB performed individual experiments; PS, JW, MBF and LM provided ideas, animal tissue and analysis. All authors reviewed the manuscript.

## Supplementary Material

Additional file 1 **Supplementary Figure 1 – Expression and quantification of α-synuclein in MES23.5 cells**. Recombinant, human α-synuclein (aSyn) (A) and lysates of MES23.5 cells expressing αS (B-C) were analyzed by ELISA [hSA2/Biotinylated Syn-1]. **(A) **A typical standard curve of serial dilutions of recombinant αS (mean of triplicate wells). **(B) **Following generation of MES-aSyn cells with three different concentrations of human *SNCA *cDNA (μg/10 cm dish; total DNA transfected per dish, 5.5 μg), lysates were analyzed in 4 different dilutions, as indicated. Results show the mean absorbance signals of triplicates from a representative experiment. **(C) **Linear relationship between amount of *SNCA*-encoding cDNA transfected into MES23.5 cells and αS protein expression, as monitored by ELISA. Each lysate was analyzed in 4 different dilutions in triplicate. Linear regression of cDNA-to-protein was performed; results represent the mean aSyn values (μg) expressed from a representative experiment. Click here for file

Additional file 2 **Supplementary Figure 2 – Characterization of affinity-purified antibodies to α-synuclein using genotyped mouse brain**. Whole brain extracts of genotyped mice were generated by lysis buffer that contained NP-40 and protease inhibitors [37]; increasing amounts of the NP-40 extract (μg/lane) were loaded onto SDS/PAGE gels under reducing conditions. Immunoblots were probed with polyclonal, affinity-purified anti-aSyn, hSA-2 (top panel) and mSA-1 (third panel). Loading controls showing IgG heavy chains are shown for both blots. Lysates were prepared from from *snca *knock-out mice without a transgene (no transgene; left half) and mice that carry a human, wild-type *SNCA *transgene (*SNCA *transgene; right half; brains kindly provided by Dr. Bob Nussbaum, UCSF). Note the specific detection of full-length aSyn (16 kDa), and of 12.5 kDa and ~10 kDa truncated species of aSyn in *SNCA*-expressing mice. Click here for file
