# Supplementary material for: Cathepsin D expression level affects alpha-synuclein processing, aggregation, and toxicity in vivo
Source: Mol Brain. 2009 Feb 9;2:5. doi: 10.1186/1756-6606-2-5 (PMC2644690; doi:10.1186/1756-6606-2-5)
Supplement: Additional file 1 — Supplementary Figure 1 – Expression and quantification of α-synuclein in MES23.5 cells. Recombinant, human α-synuclein (aSyn) (A) and lysates of MES23.5 cells expressing αS (B-C) were analyzed by ELISA [hSA2/Biotinylated Syn-1]. (A) A typical standard curve of serial dilutions of recombinant αS (mean of triplicate wells). (B) Following generation of MES-aSyn cells with three different concentrations of human SNCA cDNA (μg/10 cm dish; total DNA transfected per dish, 5.5 μg), lysates were analyzed in 4 different dilutions, as indicated. Results show the mean absorbance signals of triplicates from a representative experiment. (C) Linear relationship between amount of SNCA-encoding cDNA transfected into MES23.5 cells and αS protein expression, as monitored by ELISA. Each lysate was analyzed in 4 different dilutions in triplicate. Linear regression of cDNA-to-protein was performed; results represent the mean aSyn values (μg) expressed from a representative experiment. [file 1756-6606-2-5-S1.ppt]

## Slide 1
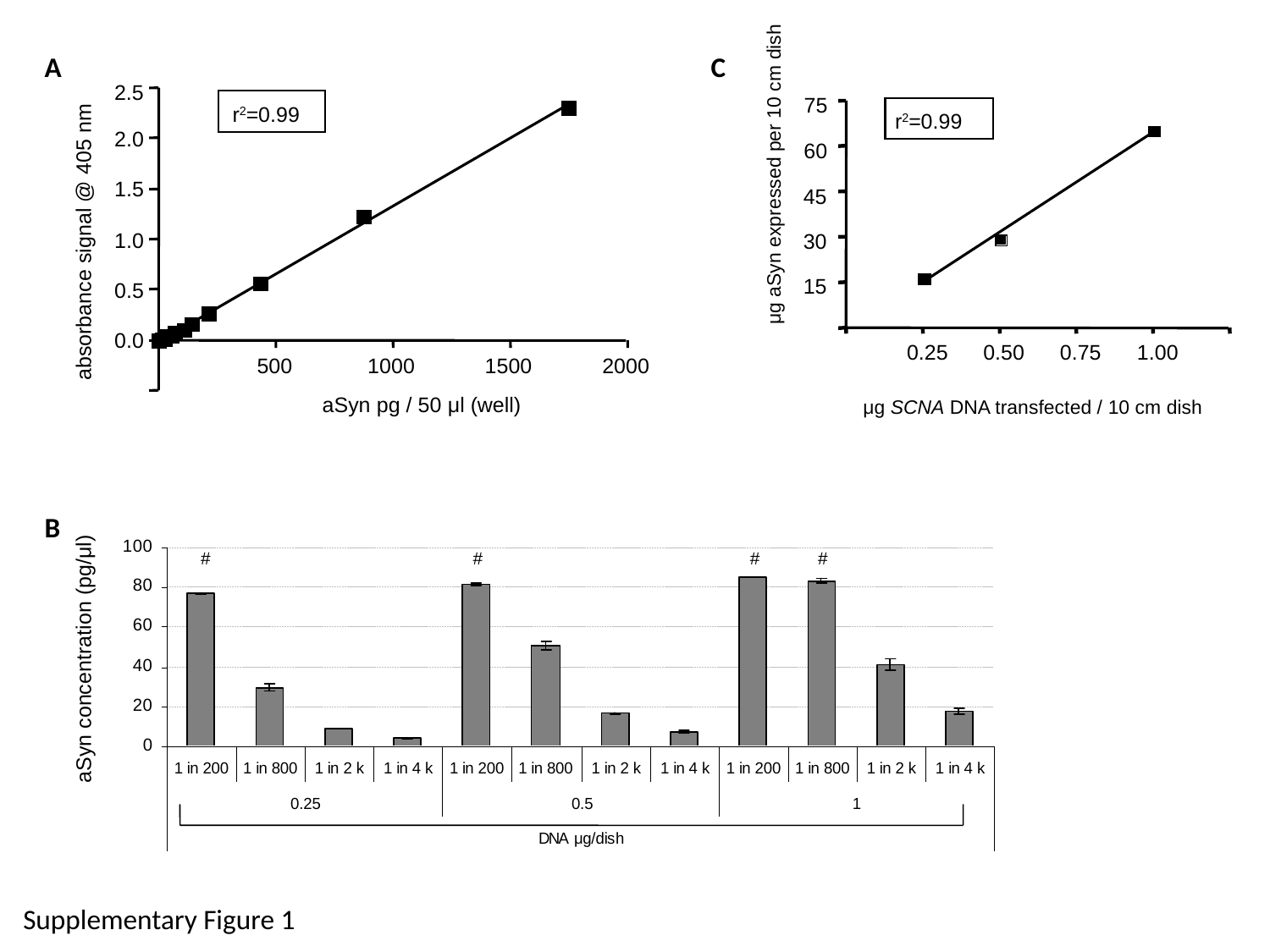

A
C
2.5
 r2=0.99
2.0
1.5
1.0
0.5
0.0
500
1000
1500
2000
aSyn pg / 50 μl (well)
75
60
45
30
15
r2=0.99
μg aSyn expressed per 10 cm dish
absorbance signal @ 405 nm
0.25
0.50
0.75
1.00
μg SCNA DNA transfected / 10 cm dish
B
# # # #
 aSyn concentration (pg/μl)
Supplementary Figure 1
