# Supplementary figures and images for: Cathepsin D expression level affects alpha-synuclein processing, aggregation, and toxicity in vivo
Source: Mol Brain. 2009 Feb 9;2:5. doi: 10.1186/1756-6606-2-5 (PMC2644690; doi:10.1186/1756-6606-2-5)

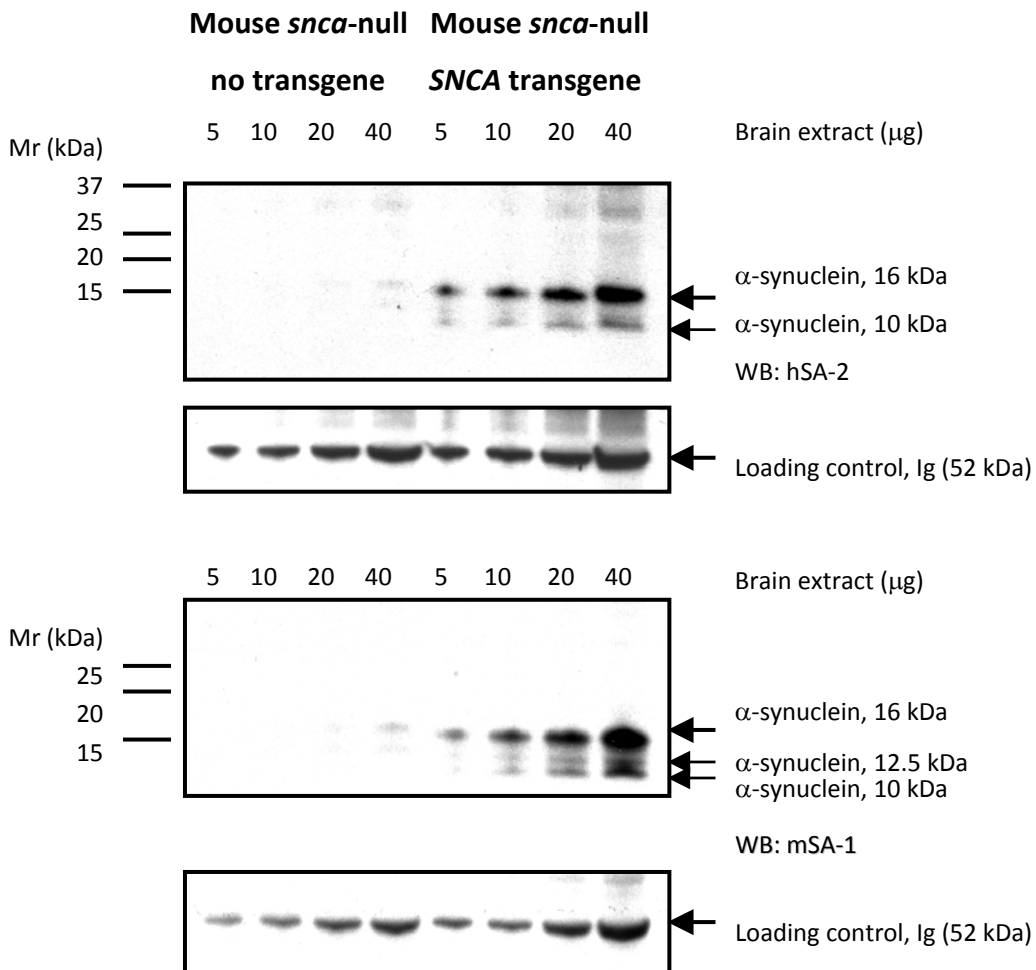

Supplementary Figure 2

Supplement: Additional file 2 — Supplementary Figure 2 – Characterization of affinity-purified antibodies to α-synuclein using genotyped mouse brain. Whole brain extracts of genotyped mice were generated by lysis buffer that contained NP-40 and protease inhibitors [37]; increasing amounts of the NP-40 extract (μg/lane) were loaded onto SDS/PAGE gels under reducing conditions. Immunoblots were probed with polyclonal, affinity-purified anti-aSyn, hSA-2 (top panel) and mSA-1 (third panel). Loading controls showing IgG heavy chains are shown for both blots. Lysates were prepared from from snca knock-out mice without a transgene (no transgene; left half) and mice that carry a human, wild-type SNCA transgene (SNCA transgene; right half; brains kindly provided by Dr. Bob Nussbaum, UCSF). Note the specific detection of full-length aSyn (16 kDa), and of 12.5 kDa and ~10 kDa truncated species of aSyn in SNCA-expressing mice. [file 1756-6606-2-5-S2.pdf]
